# Lead Environmental Pollution and Childhood Lead Poisoning at Ban Thi Commune, Bac Kan Province, Vietnam

**DOI:** 10.1155/2018/5156812

**Published:** 2018-11-18

**Authors:** Doan Ngoc Hai, Lo Van Tung, Duong Khanh Van, Ta Thi Binh, Ha Lan Phuong, Nguyen Dinh Trung, Nguyen Duc Son, Hoang Thi Giang, Nguyen Minh Hung, Pham Minh Khue

**Affiliations:** ^1^National Institute of Occupational and Environmental Health, Hanoi, Vietnam; ^2^Haiphong University of Medicine and Pharmacy, Haiphong, Vietnam; ^3^Ministry of Science and Technology, Hanoi, Vietnam

## Abstract

Lead poisoning is a public health problem in many areas of the world. Children are at particularly high risk for adverse effects of lead exposure; even at low concentrations, lead can affect physical, mental, and behavioral development. Children living near lead-zinc mines are at high risk for environmental lead poisoning, especially the contaminated soil. We conducted a cross-sectional descriptive study in Ban Thi Commune, northern Vietnam. 195 children (92,9% participation) aged 3-14 years old (average: 7.69 ± 2.90) were randomly selected from a list of all children prepared by the village health collaborators. 109 (55.90%) were boys and 86 (44.10%) were girls. The research measures were the lead concentration in native soil and the children's total blood lead concentration determined by the inductively coupled plasma-mass spectrometry (ICP-MS) method. The results showed that lead content in soil was many times higher than American Environmental Protection Agency and Vietnam standards (average 2980.23 ± 6092.84 mg/kg dry weight of soil (range 80.05 – 33820.62)). Average blood lead levels for children were 15.42 ± 6.45 *μ*g/dL (95% CI: 14.50 -16.33 *μ*g/dL). The percentage of children with lead levels >10 *μ*g/dL (value considered to be lead poisoning for children according to the Ministry of Health of Vietnam) was 79.49% of the total number of children. None of the children in this study had blood lead level (BLL) that required chelation treatment according to Vietnam MOH guideline (BLL ≥45 *μ*g/dL). There is weakly evidence that lead exposure relates to the physical development of children. Children with low lead concentrations (less than 10 *μ*g/dL) had height and weight of 1.47-3.51 cm and 1.19-2.81 kg, greater than those with BLL >10 *μ*g/dL (p>0.05).

## 1. Introduction

Lead is a heavy metal with high toxicity and harmful effects on human health. Due to its widespread use, lead poisoning is still a public health issue of concern, particularly in developing countries [1].

People are exposed to lead poisoning from a variety of sources such as industrial production (mining, metallurgy, battery manufacture, and recycling), using leaded gasoline, lead paint, electronic waster, lead-contaminated food and water, and some traditional medicines [2].

Children who are more likely to be exposed to and affected by lead poisoning are more serious cases than adults due to the specific physiological and behavioral characteristics of the developing organism [3, 4]. Children have the habit of putting hands, toys, and strange objects into the mouth. In addition, children breathe in the air close to the ground having more dust and lead than the above-the-ground air. Children absorb a greater amount of lead through the digestive tract than adults due to the incomplete digestive system, or digestive disorders and iron and calcium deficiency. Lead easily crosses the blood-brain barrier and damages the immature nervous system of the child.

There is no safe exposure threshold for children [5, 6]. Severe lead poisoning is initially characterized by lethargy, abdominal pain, loss of appetite, and irritation and then causes seizures and coma [4, 7]. Low-dose lead can lead to mental and physical development disorders, neurological disorders, decreased hem synthesis and anemia, increased acoustic feedback threshold, and decreased Vitamin D levels in the blood. The neuroleptic effects of lead on children's bodies continue as the level of lead in the blood decreases [8].

Children living near mining sites are at high risk of lead exposure due to environmental pollution. In addition, the risk of lead poisoning is due to the low socioeconomic status and the professional factors of family members [9]. In Zamfara, Nigeria, gold mining has polluted the environment, leading to the deaths of 400 children of lead poisoning [10]. Mean blood lead level of children living at Yatagan coal mine, Turkey, was 33.8 ± 15.6 *µ*g/dL in females and 38.8 ± 16.0 *µ*g/dL in males. The blood lead level was found to be >10 *µ*g/dL in 95.7% and >20 *µ*g/dL in 87.6% of all children [11]. According to Kunming's Center for Lead Poisoning Prevention (China), an average of 50-60% of children under 14 in Yunnan's mining intensive regions suffer from lead poisoning [12].

The mountainous region of North Vietnam has many lead mines. Cho Dien Pb-zinc mine locating at Ban Thi Commune, Bac Kan Province, was one of the biggest mines in Vietnam and has been mined for decades. The study by Hien NTT et al. [13] found that due to the contaminated soil environment with an average lead content of 689-1043 mg/kg (315-1870 mg/kg), hair lead content for the boys was 121.8 *μ*g/g and for the girl was 60.62 *μ*g/g. However, hair lead concentration may reflect environmental pollution but is less likely to have clinical value than blood lead concentration [14–16]. Further research on childhood lead poisoning in Ban Thi Commune is needed.

## 2. Materials and Methods

### 2.1. Setting

**Figure 1:**
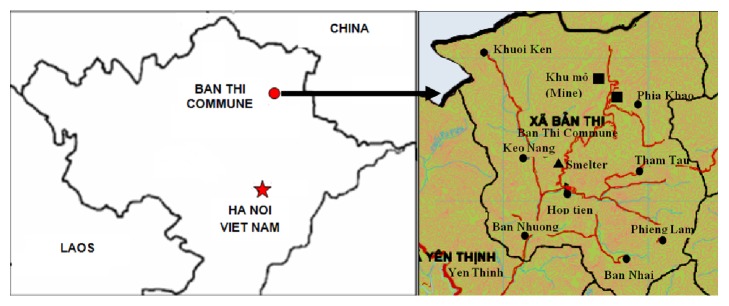
Map of Ban Thi Commune.

Ban Thi Commune, Cho Don District, Bac Kan Province is located in the northern mountainous region of Vietnam. There has a lead-zinc mine that is considered Vietnam's largest mine and has been mined for many decades. The mine is located in the north of the commune. The commune has 8 villages: Phia Khao located near the mining areas, Ban Nhuong, Hop Tien, Keo Nang located approximately 7 -10 km downstream, and other villages (Tham Tau, Phieng Lam, Ban Nhai, and Khuoi ken) with hydrogeological and geochemical contamination by metals (Figure 1). The study was implemented in 2016, which is a part of national-level research project named “Study on Lead Poisoning of Vietnamese Children and Evaluating the Effectiveness of Interventions.” All study procedures were approved by the institutional review boards at Ministry of Science and Technology and National Institute of Occupational and Environmental Health (NIOEH) of Vietnam Ministry of Health.

### 2.2. Study Sample

The list of all children aged 3-14 years of the commune was prepared by the village health collaborators. Researchers coordinated with the head of the commune health station to select randomly 210 children from the list of 250 children and prepared writing invitations for each child. Commune health staff visited family homes to describe the study and invite participation. Interested families completed an adult consent and child process.

The result study sample included 195 children (92,9% participation) aged 3-14 years (average: 7.69 ± 2.90), in which 109 (55.90%) are boys and 86 (44.10%) are girls. Of the 195 children, 86 (44.10%) children have parent involved in lead mining.

The number of children by village is as follows: 27 (13,84%) children in Phia Khao, 42 (21.53%) children in Ban Nhuong, 91 (46.67%) children in Hop Tien, 19 children in Keo Nang (9.74%), and 16 (8.20%) children from other villages (Tham Tau, Ban Nhai, Phieng Lam, and Khuoi Ken).  Table 1 shows detailed number of sampled children who participated in the study.

### 2.3. Blood Collection and Analysis

A blood volume of 1.5-2 ml was taken from each child. Blood sampling was done at Ban Thi Commune Health Station. To prevent external contamination, venipuncture site was meticulously cleaned by alcoholic cotton. Each blood sample was collected in an EDTA tube and transported to the NIOEH laboratory in dry ice packs. In laboratory, samples were stored in refrigerator at – 20°C until analysed.

Blood samples were frozen until reaching room temperature and diluted at 1: 25 in the diluent solution (0.2% (v/v) nitric acid, 0.05% w/v Triton-X-100). After dilution, samples were mixed well by vortex and centrifuge. Lead concentration in blood was measured by inductively coupled plasma-mass spectrometry (ICP/MS NexION 350X-Perkin Elmer; LOD: 0.8 *µ*g/dL; recovery: 89.7-101.2% (ICP/MS ELAN 900-Perkin Elmer); detection limit of Pb is 0.0001 mg/L).

### 2.4. Physical Measurement

Height and weight were measured by mechanical weight and height scale (RGZ–120) before collecting blood sample with accuracy of 0.1 cm in height and 0.1 kg in weight. The result was noted in health examination card.

### 2.5. Soil Collection and Analysis

The soil samples were identified at the closest 5 villages to the mine where the number of children participating in the study was the highest. Surface soil samples (1.0–5.0 cm deep) were taken at 30 different locations of the commune (in the garden of the family or from the road). The weight of the soil taken is 300 g. All soil samples were stored in polyethylene bags and transported to the NIOEH Lab. Soil lead concentration was analyzed by inductively coupled plasma-mass spectrometry (ICP/MS ELAN 900-Perkin Elmer). Soil samples were dried at room temperature (approximately 30°C), crushed, and sieved through a 0.15 mm mesh sieve. Then, 0.25 g of the sample was digested using aqua regia (HCl/HNO3=3:1) by a microwave digestion method [17].

### 2.6. Statistical Analysis

The data was imported and analyzed using SPSS software version 19.0. We used algorithms for descriptive statistics (percentage, mean, median, standard deviation, etc.) to describe the sociological characteristics of the participants. We used test chi squared when comparing two percentages and* t*-test when comparing two means. All analyses were carried out in the Faculty of Public Health, Haiphong University of Medicine and Pharmacy. The level of significance was set at a p value of less than 0.05.

### 2.7. Ethics

All study procedures involving human subjects were approved in advance by institutional review boards at the Vietnam National Institute of Occupational and Environmental Health (DTDLCN-48/15/01). All children who participated in the survey were accompanied by parents or legal guardians who were all informed and signed the consent of participating in the survey on behalf of the child.

## 3. Results

Table 2 shows that the average lead content in the soil was 2980.23 ± 6092.84 mg/kg. The highest lead soil content was in Hop Tien village (5863.11 ± 10010.78 mg/kg) and Ban Nhuong village (2759.80 ± 2375.26 mg/kg), and the lowest was in Tham Tau village (587.87 ± 491.66 mg/kg). There was a large variation in lead soil content in Hop Tien village, as one sample was taken near the furnace and ore gathering area, with very high levels of lead (33820.60 mg/kg).

**Figure 2:**
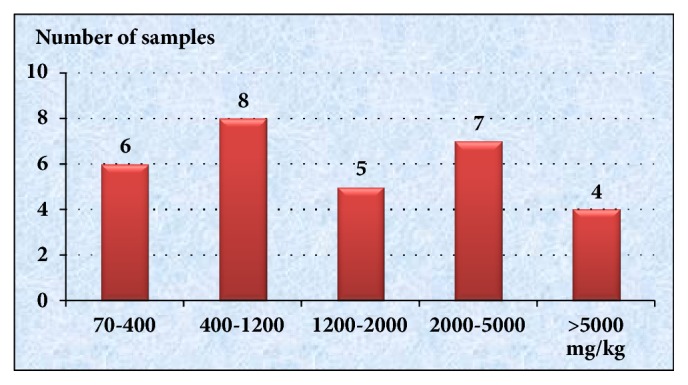
Soil sample distribution by lead level.

All soil samples have lead content in excess of Vietnam's standards (70 mg/kg for resident soil) comparing to the reference blood lead level established by EPA, and 80% (24/30) samples had PbS exceeding EPA form lead content in children's play area, of which 8 samples have lead content of 400-1200 mg/kg, 5 samples of 1200- 2000 mg/kg, 7 samples of 2000-5000 mg/kg, 4 samples of >5000 mg/kg (Figure 2).

The mean blood lead level for children was 15.42 ± 6.45 *μ*g/dL (95% CI: 14.50-16.33). The percentage of children with blood lead levels >10 *μ*g/dL (which was considered as a lead poisoning value for children as Vietnam MOH) was 79.49% of total children.

Children living in different villages had different blood lead concentration (p = 0.002). The highest BLL was in Phia Khao village with an average blood lead concentration of 23.62 ± 6.66 *μ*g/dL (95% CI: 20.98 - 26.26 *μ*g/dL) and Keo Nang village with an average blood lead concentration of 18.11 ± 5.11 *μ*g/dL (95% CI: 15.65 - 20.58 *μ*g/dL). Other areas have similar blood lead levels (Table 3).

Table 4 shows that the blood lead level was decreased according to the age group: BLL of children aged 3-5 years (nursery school age) is 16.90 ± 6.74 *μ*g/dL and for children aged 6-10 years (primary school age) is 15.31 ± 6.52 *μ*g/dL. BLL of children aged 11-14 years (secondary school age) was 13.92 ± 5.58 *μ*g/dL (p >0.05). Mean BLL of boys is 16.53 ± 5.95 *μ*g/dL, which is higher than girls (14.01 ± 6.80 *μ*g/dL, p < 0.01).

Children who live with mother or father involving in lead mining have significant lower blood lead level than others (p < 0.01). There was no statistically significant difference in the percentage of children with blood lead >10 in the group with no relatives exposed to lead, compared to the control group (p >0.05).

In the same age group, the height and weight of children with blood lead levels ≤10 *μ*g/dL were higher than those of children with blood lead levels >10 *μ*g/dL, but there was no statistically significant difference (p>0.05) (Table 5).

## 4. Discussion

Lead exists naturally in soils at levels of 10 to 50 parts per million (ppm). Higher levels may indicate lead contamination. Because of human-caused lead additions to soil, urban and residential soils often have higher lead levels than “native” soils [18]. Areas near existing or former smelters, tailings from metal ore mines, fossil fuel-fired electrical power plants, or cement factories often have elevated soil lead levels.

In our study, the average lead content of soil samples was 2980.23 ± 6092.84, 42 times higher than Vietnam's standard (70 mg/kg) and several times higher than EPA's standard (1200 mg/kg) (Table 1).

Different area of the commune had different soil lead concentration. Near the mining site, Phia Khao village, the soil lead content was 1930.05 ± 1611.11 mg/kg. But in Hop Tien and Ban Nhuong, the content of lead is still higher (Table 2). That can be explained by the fact that although these two villages are located about 7-10 km from the mine, there are some lead ore transportation routes to the gathering area, sorting, and ore sorting in the two villages.

The research conducted by NN Hien et al. [13] showed that the concentration of lead in Phia Khao soil was 1043 mg/kg (166-1870) and in Ban Nhuong and Hop Tien 781 mg/kg (113.5-1578.0) and 689 mg/kg (315.5-928.0). This level is lower than that of our study, which can be explained by the fact that the research conducted by N TT Hien was five years earlier than our study; during that time, lead in the soil could be accrued by lead mining and production activities.

Figure 2 showed that 100% samples had lead content exceeding Vietnam standard and 80% (24/30) samples had PbS exceeding EPA for lead content in children's play areas. Taking the reference blood lead level established by the US Centers for Disease Control and Prevention (CDC) in 1991 of 10 *μ*g/dL, EPA has set a standard for lead content in children's play areas not to exceed 400 mg/kg. With higher soil lead levels in Ban Thi Commune, the average blood lead level of children can be expected to be higher than 10 *μ*g/dL (reference value, CDC, 1991, Vietnam MOH 2012) [5, 19]. In fact, according to our research, the average blood lead level for children in Ban Thi was 15.42 ± 6.45 (95% CI 14.50-16.33 *μ*g/dL). The percentage of children with blood lead levels >10 *μ*g/dL was 79.49%. None of the children in this study had BLL that required chelation treatment according to Vietnam MOH's guideline (BLL≥45 *µ*g/dL) [19]. The BLL of children in our study is similar to that in Von Schirninding's study on children living near the lead mine of Aggeneys in South Africa (mean BLL = 16.5 *μ*g/dL) [20] but higher than that of the study on BLL of children living in Ranjana (mean BLL: 7.7 *μ*g/dL for boys and 8.56 *μ*g/dL for girls) [20].

Compared with other studies in Vietnam, the average BLL of children in this study was lower than the average blood lead level of children in the lead recycling village of Hung Yen [21, 22] but higher than that in children in Ho Chi Minh City [23]. In the lead recycling village of Hung Yen, recycling facilities was located in the residential areas and almost used the backward handicraft technology; therefore, environmental pollution is more serious than that of Ban Thi Commune. Meanwhile, there was no lead mine or lead recycling facilities in Ho Chi Minh City; that is why the risk of lead exposure in children is lower.

Children living near the mine (Phia Khao village) had the highest mean blood lead level (23.62 ± 6.66 *μ*g/dL), followed by children living in Keo Nang (18.11 ± 5.11 *μ*g/dL). In other areas, children have similar blood lead levels. Although the level of lead in the soil in the vicinity of the mine is not higher than that in other areas of the commune, children living near the mine may have low socioeconomic status, which is a high risk group for lead exposure [9]. In Hop Tien village, the average lead content was higher than that in other places due to the large variation in lead content in the soil samples; especially, there was one soil sample collected near the furnace and ore gathering area that had the content lead of 33820.62 mg/kg, but the residents have already known that problem and restricted children from having fun at this area; the risk of lead exposure therefore is not higher than other areas.

The younger child has the higher risk of lead exposure due to the behavioral physiology of the child. Young children often play on the ground or have a habit of putting objects in their mouths and do not yet form good personal hygiene habits (washing their hands before eating), so dust and soil containing lead from foreign objects will enter the digested tract and be absorbed into the blood. Table 2 shows that the mean blood lead concentration of children aged 3-5 years was 16.90 ± 6.74 *μ*g/dL, higher than that of the age group of 6-10 years (15.31 ± 6.52 *μ*g/dL) and statistically higher in comparison with children aged 11-14 years (13.92 ± 5.58 *μ*g/dL) (p <0.05).

Blood lead levels are also different between boys and girls. BLL of boys was significantly higher than that of girls (16.53 ± 5.95 and 14.01 ± 6.80; p <0.05). This trend is similar to that of Chinese children [24].

Children living with their parents who are engaged in mining had lower blood lead levels than other children (p = 0.007). The results show that parent occupational factors do not play an important role in lead poisoning of children, but primarily environmental factors do. Families who are engaged in lead production may have better economic conditions and better conditions to care for their children, which in turn limits their children's exposure to lead.

The abovementioned results help to guide the prevention of lead poisoning in children in Ban Thi. Intervention should be giving priority to children, especially children under 10 years of age, and interventions should focus on raising awareness among parents and caregivers about lead poisoning and prevention. Remedying lead pollution is a top priority.

One of the effects of low-dose lead exposure is that it affects the physical development of the child. Research by Kafourou et al. [25] showed that when the blood lead level increased to 10 g/dL, the head circumference decreased by 0.33 cm, the height reduced by 0.86 cm, and the chest circumference reduced by 0.40 cm. Little B.B. (2009) study of children in Dallas, Texas, found that in 2002 the average height and weight of children were 4.5 cm and 4.0 kg greater than in 1980 [26]. The average BLL of children in 1980 was 23.6 *μ*g/dL, much higher than that of 2002 (1.6 *μ*g/dL). Children with low blood lead levels had a height, weight, and BMI of 3.9 cm, 3.5 kg, and 1.1 units, respectively. Our study showed that children with low lead concentrations (less than 10 *μ*g/dL) had height and weight 1.47-3.51 cm and 1.19-2.81 kg greater than those with BLL >10 *μ*g/dL. Lead exposure has a detrimental effect on the physical development of children.

## 5. Conclusion and Recommendation

Lead mining and smelting activities have resulted in environmental pollution in the residential area near the mine. The study result showed that 80% (24/30) samples had PbS exceeding EPA form lead content in children's play area and 79,49% of children living in Ban Thi Commune had BLL > 10 *μ*g/dL.

It is important to consider and implement interventions to minimize the impact of child health on the community and to conduct research on the environmental status and health of children living near other lead-zinc mining sites in Vietnam.

## Figures and Tables

**Table 1:** Characteristics of the sampled inhabitants according to age and sex.

Locations	Mean age (SD)	Number of participants		Total
		Boy	Girl	
Ban Nhuong	7.90 ± 3.23	25	17	42
Hop Tien	7.35 ± 2.90	53	38	91
Keo Nang	8.95 ± 1.61	10	9	19
Phia Khao	7.81 ± 3.15	16	11	27
Others (TT, KN, BN, etc.)	7.31 ± 2.60	5	11	16
Total	7.69 ± 2.90	109	86	195

**Table 2:** Lead concentration in soil samples (mg/kg).

Village	N	Median	Mean ± Std. Dev.	Min.	Max.
Phia Khao	5	1042.39	1930.05 ± 1611.11	737.18	4562.84
Ban Nhuong	5	3093.74	2759.80 ± 2375.26	80.05	6288.74
Hop Tien	10	2390.36	5863.11 ± 10010.78	110.73	33820.62
Keo Nang	5	1059.89	877.44 ± 525.64	138.88	1397.02
Tham Tau	5	261.98	587.87 ± 491.66	211.45	1213.75
Total	30	1214.35	2980.23 ± 6092.84	80.05	33820.62

**Table 3:** Blood lead level (BLL) of children by villages (*μ*g/dL).

Village	n	Mean ± SD	Geo. Mean	(95% CI)	No. (%) >10 *µ*g/dL
Phia Khao	27	23.62 ± 6.66	22.71	20.98 - 26.26	27 (100.00)
Keo Nang	19	18.11 ± 5.11	17.37	15.65 - 20.58	18 (94.74)
Ban Nhuong	42	13.51 ± 4.91	12.82	11.98 - 15.04	32 (76.19)
Hop Tien	91	13.43 ± 4.76	12.66	12.44 - 14.42	68 ( 76.40)
Others	16	14.65 ± 7.88	12.75	10.45 - 18.85	10 (62.50)
Total	195	15.42 ± 6.45	14.20	14.50-16.33	155 (79.49)

**Table 4:** Children blood lead level among studied children (*µ*g/dL).

Characteristic	n	Mean ± SD	Geo. Mean	(95% CI)	No. (%) >10 *µ*g/dL
Age (years)					
3-5	45	16.90 ± 6.74	15.69	14.88-18.93	39 (86.67)
6-10	113	15.31 ± 6.52	14.07	14.10-16.53	88 (79.28)
11-14	37	13.92 ± 5.58	12.95	12.06-15.78	28 (75.68)

p value		0.109			0.28

Sex					
Boy	109	16.53 ± 5.95	15.49	15.39-17.66	94 (86.24)
Girl	86	14.01 ± 6.80	12.73	12.55-15.47	61 (70.93)

p value		0.006			0.009

Parent involvement in lead mining					
No	109	16.51 ± 7.18	14.99	15.15-17.87	90 (82.57%)
Yes	86	14.03 ± 6.92	13.26	12.94-15.12	65 (75.58%)
p value		0.007			0.23

**Table 5:** Relationship between physical growth and blood lead level among children.

Age (years)	**BLL≤10 **µ**g/dL**			**BLL>10 **µ**g/dL**			p
	N	Mean ± SD	95% CI	n	Mean ± SD	95% CI	
**Body Height (cm)**							
3-5	6	104.83 ± 7.88	96.56-113.11	39	101.32 ± 7.71	98.82-103.82	0.47
6-10	24	124.44 ± 10.46	120.02-128.85	86	122.97 ± 10.39	120.74-125.19	0.529
11-14	9	146.78 ± 12.04	137.52-156.03	28	143.96 ± 9.38	139.79-146.58	0.45
**Weight (kg)**							
3-5	6	16.75 ± 3.24	13.35 ± 20.15	39	14.93 ± 2.05	14.27 ± 15.60	0.58
6-10	24	24.39 ± 7.37	21.28 ± 27.50	86	23.20 ± 6.23	21.87 ± 24.54	0.429
11-14	9	37.94 ± 11.15	29.38 ± 46.51	27	35.57 ± 11.04	31.20 ± 39.93	0.38

## Data Availability

The EXCEL/SPSS data used to support the findings of this study are available from the corresponding author upon request.

## Abbreviations

BLL: Blood lead level
BMI: Body Mass Index
CDC: US Centers for Disease Control and Prevention
EPA: US Environmental Protection Agency
ICP/MS: Inductively coupled plasma-mass spectrometry
MOH: Ministry of Health
NIOEH: Vietnam National Institute of Occupational and Environmental Health
WHO: World Health Organization.
